# Influence of SGLT1 Sugar Uptake Inhibitors on Water Transport

**DOI:** 10.3390/molecules28145295

**Published:** 2023-07-08

**Authors:** Marko Sever, Franci Merzel

**Affiliations:** 1 Theory Department, National Institute of Chemistry, Hajdrihova 19, 1000 Ljubljana, Slovenia; marko.sever@ki.si; 2 Faculty of Pharmacy, University of Ljubljana, Aškerčeva 7, 1000 Ljubljana, Slovenia

**Keywords:** molecular dynamics simulations, SGLT1 protein, water permeation, inhibitors, intrinsic domain motion, principal component analysis

## Abstract

Sodium glucose cotransporters (SGLTs) are cotransporters located in the cell membrane of various epithelia that uptake glucose or galactose and sodium into the cell. Its founding member, SGLT1, represents a major pharmaceutically relevant target protein for development of new antidiabetic drugs, in addition to being the target protein of the oral rehydration therapy. Previous studies focused primarily on the transport of substrates and ions, while our study focuses on the effect of water transport. SGLT1 is implicated in the absorption of water, yet the exact mechanism of how the water absorption occurs or how inhibitors of SGLT1, such as phlorizin, are able to inhibit it is still unclear. Here we present a comprehensive study based on molecular dynamics simulations with the aim of determining the influence of the energetic and dynamic properties of SGLT1, which are influenced by selected sugar uptake inhibitors on water permeation.

## 1. Introduction

Efficient transport of biologically relevant polar molecules, such as water across the nonpolar lipid membrane is of paramount importance to human physiology. Because of the low expression of the facilitating water channel protein aquaporin in the small intestine, SGLT1, has been shown to be involved in water uptake [1].

SGLT1 is a symporter for glucose, galactose, and sodium ions that occurs mainly in the small intestine and kidney and is encoded by the SLC5A1 gene. The importance of SGLT1’s transport capacity for water is highlighted by mutations of the SLC5A1 gene that result in a genetic disorder of glucose-galactose malabsorption. The disease is characterized by the occurrence of severe diarrhea in newborns that is fatal if glucose and galactose are not removed from the infant’s diet [2]. The importance of water transport is also highlighted by the use of oral rehydration solutions (ORS) as the primary treatment for acute diarrhea in children. The use of ORS stimulates fluid uptake through SGLT1 [3]. Recent studies have shown that inhibition of SGLT1 has an effect on ameliorating chronic constipation when subjects are administered the selective and high-affinity SGLT1 inhibitor [4]. In addition, the prototypical inhibitor phlorizin was shown to inhibit water transport by SGLT1 in a model system [5].

The mechanism of water transport through SGLT1 and various other cotransporters have been highly discussed by various groups that have presented two main hypotheses that describe the mechanism of water transport [6]: (i) passive or osmosis driven transport [7], and (ii) active or cotransport driven transport [8]. Passive transport thus relies on maintaining an open conformation of the transporter in which both gates should be open—thus these hypotheses are focused only on the local aspect of the protein structure that would have an influence on water transport. The passive transport mechanism thus relies only on continuous pathways for water to be present in addition to a sufficiently large orifice or bottleneck area for the water molecules to pass. The active cotransport-driven hypothesis relies on a stoichiometrically related cotransport of water through various proposed mechanisms and would rely on a conformational change taking place in the system, it would also implicate an absence of a continuous pathway to be present in discrete conformational states [6,7,8].

The experimental evidence is available to corroborate both of the contradictory water transport hypothesis. Zeuthen’s et al. mutagenesis study of mutating a set of residues that are thought to be lining the water channel in SGLT1 displays that by increasing the spatial size of selected residues, the water permeability increases, thus giving validity to the passive water transport hypotheses [5]. Additional experimental evidence in favor of the passive water transport in SGLT1 was provided by Erokhova et al. [9]. On the other hand, there are reports of oocytes expressing SGLT1, which instantly react and start pumping water with the addition of glucose, while without the substrate, no water transport is observed [6]. The instant reaction of the transporter, the ability of the transporter to transport water against its concentration gradient and the link of the water transport to the substrate transport corroborates the active transport hypothesis.

There is a wealth of evidence - biochemical, kinetic, and structural [10,11,12] to confirm that SGLT1 switches its conformation between the outward-facing (OF) and inward-facing state (IF), using a model called an alternating access mechanism, in which the substrate binding site is sequentially exposed to either the extracellular or intracellular side [13]. It is postulated that the LeuT-fold cotransporter SGLT1 changes its conformation via the “rocking-bundle” type motion, which is a type of alternating access mechanism. The model is based on a high degree of coordination of local gates in the substrate channel of the transporter, in addition to global conformational changes [14]. The domain organization of the cotransporter is of paramount importance in achieving the different conformational states. In terms of domain organization, transmembrane segments TM1, 2, 6 and 7 constitute the core or bundle domain, while TM3, 4, 8 and 9 constitute the scaffold or hash domain. Both primary domains are linked with TM5 and TM10 helices which form the gating domain [15,16]. It is evident that the transport of membrane proteins involves highly diverse events of structural changes, ranging from local rearrangements at the binding sites and their gating elements to global conformational transitions. It seems necessary to investigate the influence of protein dynamics on water permeation, which could best be elucidated by molecular dynamics simulations (MD) [17,18].

The influence of the dynamics of domains that interact with the water pathway has not yet been investigated in SGLT1. In this study, we focused on the modulation that the known sugar inhibitors, such as phlorizin, mizagliflozin, and sotagliflozin exert on protein dynamics upon binding and how this binding affects water permeability. We hypothesized that the effect of water transport in SGLT1 must be due to an interplay between the local (residue-based channel properties) and global (domain movement) aspects of protein structure and dynamics. Using extensive MD simulations, we investigated the water permeation of SGLT1 in different OF conformational states which approximate the apo and holo states as well as states with bound inhibitor molecules. The equilibrium dynamics of the different conformational states were used to elucidate energetic and dynamic aspects of the protein’s ability to permeate water. We demonstrate the applicability of the novel computational approach as a tool to evaluate the structural flexibility of a transporter as a determinant of molecular transport that should be targeted by inhibitors.

## 2. Results

The systems used for the MD simulation studies are based on the homology model of the OF conformation of SGLT1, obtained through personal communication from Prof. Grabe, which was extensively experimentally validated in his previous studies [19]. In addition to the apo form of the OF conformation (out-apo) and the holo form (out-gal) containing galactose bound in its binding site, we constructed three systems with bound inhibitors, phlorizin (phlori), mizagliflozin (miza), and sotagliflozin (sota), all in the OF conformation of the SGLT1 using the docking procedure as described in the Methods section. Each system was placed into a 3:1 POPC:CHOL bilayer, electroneutralized by 0.15 M NaCl and simulated up to 1 μs of total simulation time as described in the Methods section. Extensive equilibrium atomistic MD simulations of all of the generated systems were performed to attain statistical significance. The atomistic detail enabled us to probe the various factors that could influence the mechanism.

### 2.1. Water Permeation and Energetics of the Transporter

**Figure 1:**
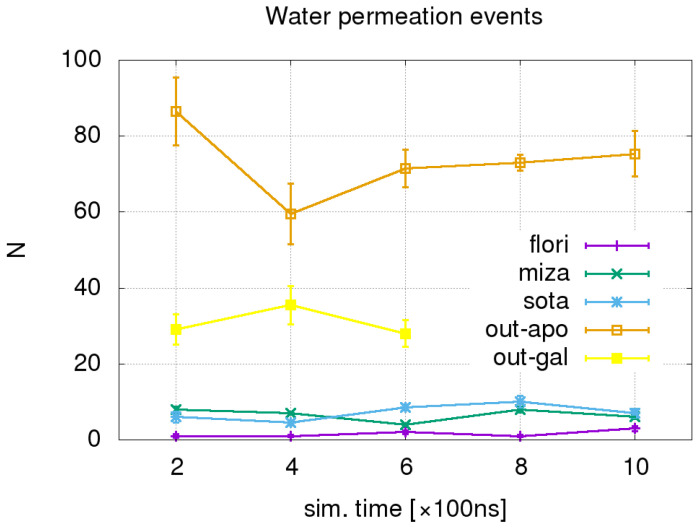
Total number of water permeation events per 100 ns along with sequential trajectory segments. The orange line denotes the OF apo conformation, yellow—the OF conformation with bound galactose, violet—the OF conformation with bound phlorizin, blue—the OF conformation with bound sotagliflozin, and green line—the OF conformation with bound mizagliflozin.

We first analyzed water permeation as a function of time in all simulated systems by counting the full permeation events in both extracellular and intracellular directions during 100 ns time intervals as explained in the Section 4. The results are shown in Figure 1.

We can clearly observe the difference in the amount of water permeation events between the systems in OF apo- and galactose-bound systems, which show high permeability, and systems with bound inhibitors, which all show significantly reduced permeability. The presence of inhibitor molecules has similar effects on water permeation in all three systems.

**Figure 2:**
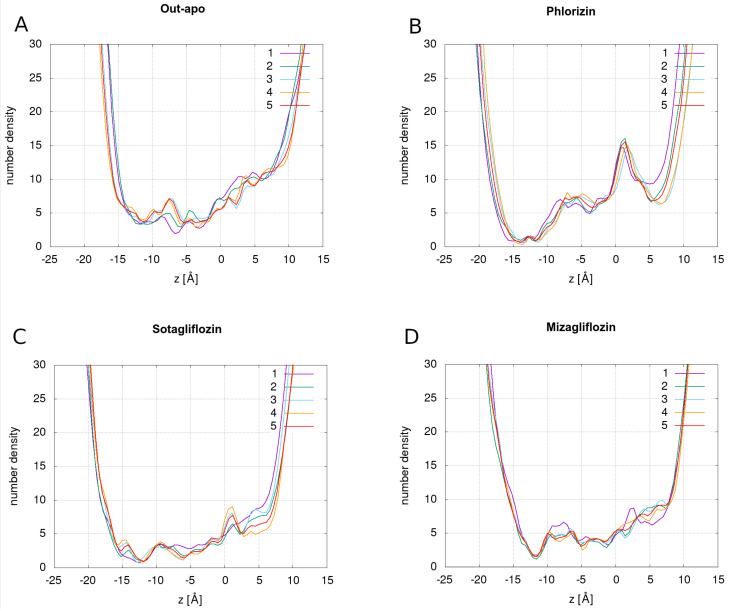
Water number density graphs of OF apo conformation (Out-apo) (**A**) and for conformations with bound phlorizin (**B**), sotagliflozin (**C**), and mizagliflozin (**D**). Water profiles are shown for five sequential segments of trajectory; each is averaged over the time span of 200 ns.

In order to screen the water distribution within the protein, we calculated the number density profiles of water as a function of *z* coordinate along the transmembrane direction. Asymmetric U-shaped water profiles are shown in Figure 2 and display minima on the intracellular side while the extracellular side remains more open. Water number density of a system without the bound inhibitors (OF apo), Figure 2A, is over its entire range slightly higher, indicating a more open channel.

We can identify three characteristic minima common in all systems. The minimum at z=5 Å corresponds to the location of the extracellular gating residues, minimum at z=−5 Å corresponds to the location of the intracellular gating residues, as also referenced in the literature. The locations of the extracellular and intracellular gating residues were deduced by calculating the z-axis position of the center of mass (CoM) of the selected residues. The most pronounced minimum at z=−12.5 Å is formed in systems with bound inhibitors, Figure 2B,C. Thus the main bottleneck area is not present in the intracellular gating residues location, as was speculated.

**Figure 3:**
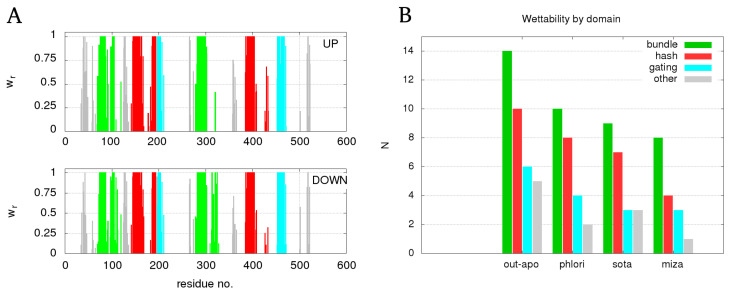
(**A**) residue wettability caused by the permeating water in extracellular (upper panel)—and intracellular direction (lower panel) for out-apo system. Bars belonging to the bundle domain are colored green, hash domain red, gating domain blue, and the rest gray. (**B**) bar chart with cumulative number of residues with wettability values beyond 0.9 decomposed over different domains.

The water profiles in Figure 2 display the distribution of all water molecules inside the protein, but this does not imply that all this water also permeates through the protein. To distinguish permeating water, we needed to select only those water molecules that traverse the entire protein length in either the extracellular or intracellular direction, which we selected as described in the Methods section. The pathways of the permeating water were then determined by referencing the protein residues that were in contact with the permeating water. In this regard, we determined the “residue wettability” $w_{r}$ as the ratio between the number of residue atoms that were found within the cutoff distance of $r_{c}=4$ Å to a permeating water molecule relative to the total number of atoms in a residue. $w_{r}=1$ corresponds to a residue with a complete overlap with spheres around moving water molecules. $w_{r}$ values of residues in out-apo protein that were found in contact with permeating water are shown in Figure 3A. Regarding the importance of the domain organization of SGLT1, we show the number of residues belonging to different domains in different colors. Figure 3B summarizes the number of residues reaching the wettability beyond 0.9 in each domain separately for all simulated systems. The lists of residues for each system are provided in Supplementary Materials.

The similarity of the graphs for the extracellular and intracellular directions of water permeation in Figure 3A suggests the same pathways are used for water transport in both directions, which is indicative for the passive water transport. The presence of inhibitors results in a smaller number of residues in full contact with permeating water relative to the out-apo system. In addition, we found that the majority of residues surrounding the water transport pathways are part of the bundle and hash domains and only a smaller fractions are a part of the gating domain, and the remaining part of the protein.

**Figure 4:**
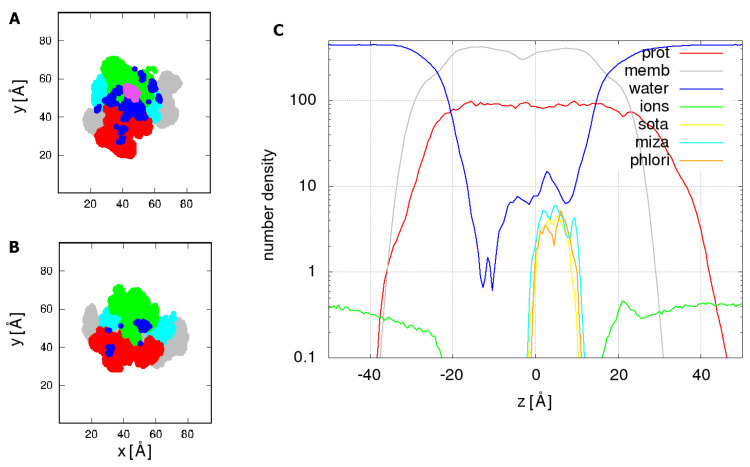
(**A**) 2D cross-section of the phlorizin system in the range, 0<z<5 Å, at the phlorizin binding site; domains are colored (bundle—green, hash—red, gating—cyan), position of phlorizin is shown in pink and water dark blue; (**B**) 2D cross-section of the phlorizin system in the range, −10<z<−5 Å. (**C**) One-dimensional density profiles in logarithmic scale of phlorizin, sotagliflozin, and mizagliflozin relative to the other components of the system: protein, membrane, water, and ions. For the sake of clarity, we show other components only for the phlorizin system.

The distribution of water within the protein was further analyzed by calculating 2D cross sections of thickness 5 Å parallel to the membrane of atom densities belonging to different parts of the system. Densities were averaged over the last 100 ns of phlorizin system simulation and are shown in Figure 4A,B. One-dimensional density profiles along the membrane normal in Figure 4C show similar distributions of all three inhibitors relative to the constitutive components of the system, suggesting a similar binding mode of inhibitors.

The significant presence of water located predominantly at the interface between the bundle and hash domains in the binding site region suggests that water permeation may not be directly hindered by steric hindrance due to an inhibitor. Surprisingly, a smaller amount of water is located on the opposite, intracellular half of the water channel distal to the inhibitor binding site.

**Figure 5:**
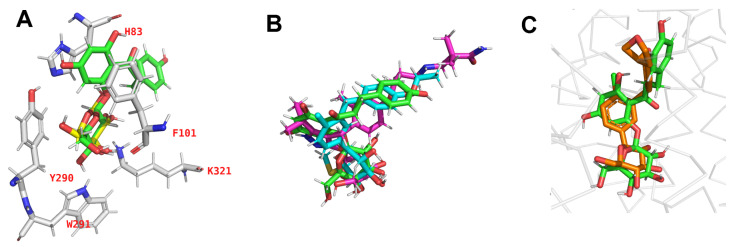
(**A**) Pose of phlorizin (green) and galactose (yellow) from in the SGLT1 binding site from simulation, with major interacting residues displayed. (**B**) Superimposed binding poses of phlorizin, sotagliflozin (cyan) and mizagliflozin (magenta), obtained by aligning the binding site residues of each system. (**C**) Superposition of binding poses of crystal structure of empagliflozin (orange) bound to homologous transporter SGLT2 (PDB id: 7VSI [20]) and the pose from the simulation of phlorizin.

The inhibitors bind and remain stable at the binding site, as shown in Figure 5A for phlorizin, whose sugar moiety overlaps with the galactose position. Superimposed binding poses of phlorizin, sotagliflozin, and mizagliflozin, obtained by aligning the binding site residues of each system, demonstrate the conservation of the sugar moiety and suggest that the inhibitors studied adopt a similar binding site position during simulation, see Figure 5B. Such a binding mode is consistent with other studies, such as in the SGLT2-empagliflozin solved structure [20], which is shown in Figure 5C along with the phlorizin position. The superposition in Figure 5C was obtained by aligning the binding site residues of both transporters.

Since the domain organization of the SGLT1 is supposed to have a profound impact on the conformational switch of the transporter from the inward facing the OF conformation and vice-versa, during the alternating access cycle, we assume that the bound inhibitors should directly affect the domain structure and its dynamics. Therefore, we compared the interaction energy of each the inhibitor molecules to the interaction energy of the natural ligand-galactose with the transporter.

The interaction energies between the selected molecules and SGLT1 were calculated by taking into account all atomic pairwise contributions of the electrostatic and van der Waals potential energy terms and are displayed in the second column of Table 1. To assess binding free energies of individual ligands, we used the end-point calculation with molecular mechanics Poisson–Boltzmann surface area (MMPBSA) method as implemented in the program MMPBSA.py [21].

We found that the inhibitor molecules demonstrate stronger binding, i.e., lower binding free energies with SGLT1, compared to the natural ligand. Such behavior is expected since the inhibitor molecule blocks the mode of action of the transporter while the natural ligand is transported by it. Thus it must remain less tightly bound. In addition, galactose is a smaller molecule than the inhibitor molecules, so the size should provide fewer interactions with neighboring residues.

Mizagliflozin turns out to have most favorable interaction energy compared to the other two inhibitors which is in line with the known values of inhibiting potency for inhibiting sugar transport in SGLT1, which are adopted from [22] and are given in Table 2.

We assumed that the inhibition of protein function should be related to the stiffening of the protein structure, caused by an increased interactions between protein subunits. Based on the results of the domain wettability analysis, in addition to the steric hindrance analysis, we found that the main domains that interact with the water flow are the bundle and the hash domain. The interaction energy between the bundle and hash domain as a function of simulation time is given in the last column of Table 2.

We observed the marked difference in the increased interaction between the bundle and the hash domain helices in cases of inhibitor binding versus the apoprotein and protein with galactose binding. Such an increased interdomain interaction could thus possibly rigidify the entire protein structure and thus decrease the water and also substrate transport through the cotransporter. The interaction between the domains remains fairly constant and stable.

**Figure 6:**
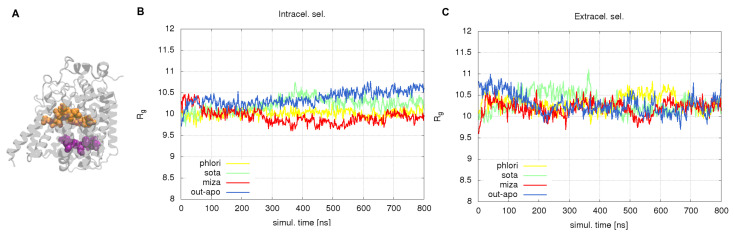
(**A**) selected layers of residues around water channel: extracellular side (orange) and intracellular side (magenta). Radius of gyration of selected group of residues: intracellular selection in panel (**B**) and extracellular selection on panel (**C**). Yellow curves correspond to phlorizin, green to sotagliflozin, red to mizagliflozin and blue to OF apo conformation.

To verify the eventual constriction effect on residues lining the water channel, we made two unique selections of residues for all systems at two cross-sectional layers of the channel corresponding to the intracellular and extracellular side of the binding side as shown in Figure 6A. We calculated the radius of gyration for both selections in all systems as a measure of their spatial extension. Inspecting the behavior of the radius of gyration for the intracellular selection, which also represents a constriction area in the water pathway, we found that the constricting effect of the inhibitor binding is noticeable and turns out to be the strongest in case of mizagliflozin, Figure 6B. The effect is somehow weaker for the extracellular selection, Figure 6C.

### 2.2. Dynamics of the Domain Structure

**Figure 7:**
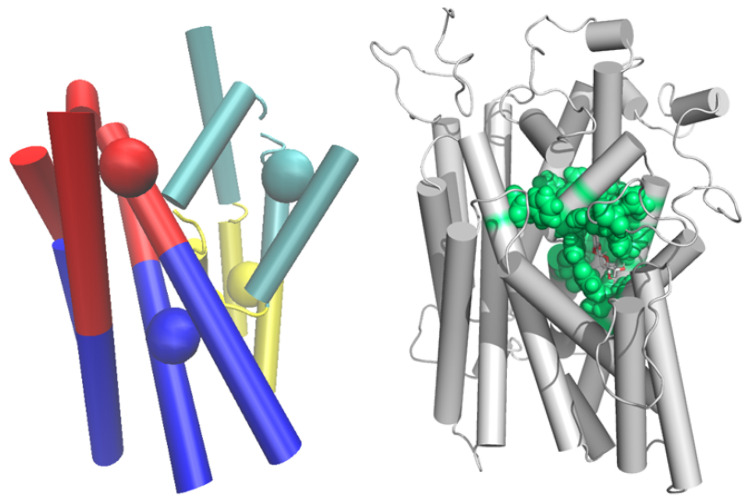
(**Left**) four-bead representation of hash (red and blue cylinders and beads) and bundle domain (green and yellow cylinders and beads). (**Right**) CoM of selected residues around binding site represented by green beads.

We assumed that the more favorable interaction between the bundle and the hash domain leads to increased stiffening or, equivalently, decreased flexibility of the protein, which should be evident in the internal dynamics of the protein. Therefore, we performed principal component analysis (PCA) on simulated trajectories and calculate dynamic flexibilities that can be easily extracted from PCA as described in the Methods section. PCA was carried out for all systems equally in two ways: (i) for the maximally coarse-grained domain structure represented by four beads, and (ii) for selected 18 residues around binding site (choices shown in Figure 7). Each selected domain (bundle and hash) was divided along the *z*-axis into two objects, where the individual TM α-helices of each domain was halved and selected into the same object used in the PCA analysis, each object is represented by a bead in the CoM of the selection with a mass corresponding to the selection. The selections were chosen in such a way that they had approximately the same mass and contained functionally important amino acid residues. Each domain thus consisted of only two objects, as depicted in Figure 7. The selection of residues was made according to proximity criterion to the binding site and CoM of individual residues were used for bead representation.

An additional parameter that should affect the ability of the transporter to permeate water is the area of the orifice at the bottleneck section of the water pathway. There is, however, no straightforward way to define it. Here we defined the channel opening as the minimum of the time-averaged number density of the water profile, such as shown in Figure 2, which we multiplied by the cross-section of a single water molecule (∼7.1 Å${}^{2}$).

The systematic domain movements which we observed in PCA analysis and which are assumed to characterize the rocking bundle motion, comprise the following three types:

(A) Alternating opening and closing of the mouth on opposite sides,
(B) Up–down sliding of one domain relative to the other along the channel direction,
(C) Opening or closing of the entire water channel simultaneously.

**Figure 8:**
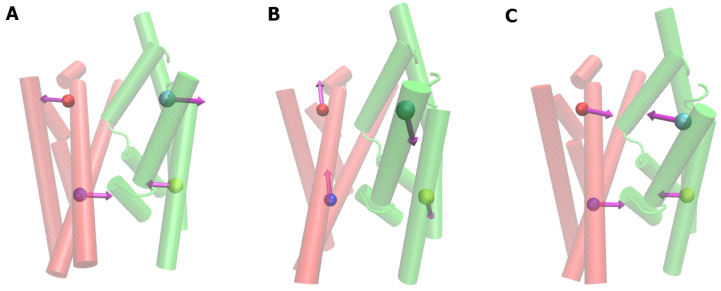
Graphical representation of the main three types of representative movements observed (hash domain—red, bundle domain—green); (**A**) alternating opening, (**B**) up-down sliding and (**C**) simultaneously opening of the channel.

We display these in Figure 8.

**Figure 9:**
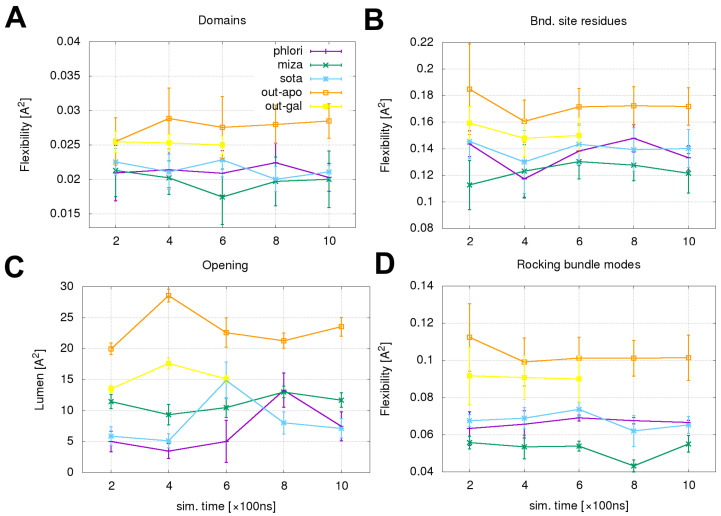
(**A)** domain flexibility per trajectory segment, (**B**) binding site residues flexibility per trajectory segment, (**C**) orifice surface in the constriction region, (**D**) flexibility of the sum of three main “rocking-bundle” type motions.

Analysis of protein dynamics was performed for all simulated systems in sequential 200 ns time intervals. Flexibilities of domain motions and selected binding site residues motions were calculated according to Equation (2) and are shown in Figure 9A,B, respectively. Both sets of results indicate significantly lower levels of dynamic flexibility present in systems with bound inhibitors relative to the OF apo system. The results of the channel opening (Figure 9C) show lower values for systems with bound inhibitors relative to the apo system meaning less favorable conditions for water permeation. The most consistent difference between the apo- and inhibitor-bound systems was found for a comparison of flexibilities associated with the three selected “rocking bundles” modes can be seen in Figure 9D. The results obtained by summing the contributions of all three selected modes shown in Figure 8, were calculated with the aid of Equation (3).

All the results of the dynamic analysis included align with the number of water permeation events displayed in Figure 1, suggesting the importance of these two factors for water transport.

## 3. Discussion

Our results suggest that the mechanism of water permeation in SGLT1 is influenced by various effects that dynamically modulate mainly passive transport. We observe that the local effect of stiffening the neighboring binding site residues propagates to the domain organization since the apo- conformation is significantly more flexible versus the inhibitor-bound systems. Since the cotransport hypothesis postulates that water transport is a function of the conformational change of the cotransporter, the domain organization certainly has an effect on the water transport since it modulates the water channel significantly in both main conformations. By reducing the mobility of domains and rigidifying the domain structure, therefore, water transport is also limited. Due to the fact that the conformations remain stable during the simulations and that we observed sporadic events of water permeation even in the systems with bound inhibitors, we can conclude that the cotransporter is not closed tightly enough to allow perfect sealing of the water pathway. It seems that an orifice is present at all times and that the water transport through the cotransporter is passive. As we see in the results, if the inhibitor binds to the cotransport, it reduces its flexibility or, in other words, rigidifies it. Yet there are big differences in the water permeation events between the OF-apo system and the bound inhibitors, so the passive water transport mechanism is modulated by the dynamics of the domain organization. The fluctuation of domains with the bound inhibitors slows the movement of the transporter. It appears that passive permeability in the transporter is not only a function of the size of the opening but also a function of the dynamic properties of the system. In addition, the behavior of the selection of characteristic movements that describe the rocking-bundle type motion is the same, the movements of the inhibited systems are rigidified. Thus the dynamic aspect of the protein structure is found to be very important in the mechanism.

## 4. Materials and Methods

### 4.1. Homology Models

As the solved structures of human SGLT1 protein were not available at the onset of our study, we used the homology model that we obtained from M. Grabe and P. Bisignano, Cardiovascular Research Institute, University of California, and was published in [19]. This homology model (residues 1-543) was constructed by matching the structural core consisting of TM1-TM10 helices with the X-ray structure of N-acetylneuraminic acid transporter from Proteus mirabilis (SiaT) (PDB id 5NV9), while loops were modeled by using vSGLT as the template (PDB id 3DH4) [19].

To validate the homology model used in our study using the recently solved structure (PDB id 7SLA) [23] of SGLT1, we performed a shorter MD simulation of the OF solved structure (OF cryoEM) (production run: 200 ns) of the truncated chain containing the entire core region of the SGLT1 (residues 19–543) following the same protocol as for the homology model, which is described in the MD simulations subsection. The missing residues (1–18) belong to the TM0 unit, which is located outside the core region, and has been omitted in our model. We calculated the root mean square deviation (RMSD) for the core region as a function of time for the OF cryoEM simulation against the time-averaged structure of the homology model over the same time period, and vice versa: RMSD of the homology model simulation against the time-averaged structure of the OF cryoEM. The mean RMSD values are 2.08 Å and 1.96 Å, respectively. Both RMSD curves are shown in Figure S1 in the Supplementary Materials.

### 4.2. Docking of Inhibitors

The docking calculations of the studied inhibitors were performed using the Biova Discovery Studio software suite [24]. The OF conformation of the SGLT1 transporter was used since it is presented in the literature, that the inhibitor molecule binds from the extracellular side to the OF conformation [19].

The model of the OF conformation of SLGT1 was prepared in a form suitable for docking, and the protonation state of the cotransporter was set to pH 7 with the use of PROPKA [25]. The structure of the inhibitors used was obtained from the PubChem [26] database in .sdf format and was inspected regarding the correct atom connectivity. We optimized the geometry of the inhibitors using Gaussian [27] with the 6-31g* basis set by energy minimization on the Hartree Fock level. A spherical docking area with a radius of 15 Å was chosen, which was centered in the area of residues, which are by mutagenesis studies confirmed to participate in the binding of phlorizin [19]. The GOLD [28] docking algorithm with its default GoldScore [29] scoring function was used. From the top 100 obtained poses, the top 10 poses were visually inspected. The final pose was chosen based on the results of the scoring function, visual inspection of the poses, and chemical intuition.

Our docking results are in line with previous work as referenced in the literature since our final chosen docking pose is corroborated with mutagenesis studies referenced in the literature [19]. The sugar moiety of the inhibitor places itself into the sugar-binding site in the SGLT1 cotransporter, while the aglycon tail of the inhibitor is placed in the extracellular vestibule.

### 4.3. MD Simulations

Explicit solvent atomistic simulation systems were prepared using the online server CHARMM-GUI [30]. The orientation of the transporter in the membrane was chosen with the use of the »Orientations of Proteins in Membrane« (OPM) server [31]. The membrane consisted of 1-palmitoyl-2-oleoyl-sn-glycero-3-phosphocholine (POPC) and cholesterol in a molar ratio of 3:1. The system was electoneutralized in 150 mM NaCl, the TIP3P [32] water model was used. We constructed various systems in OF conformation, including apo form, and complexes with galactose, phlorizin, and sotagliflozin in metagliflozin each bound in the binding site. Each system consisted of approximately 94,000 atoms and was processed separately as follows. Initial equilibration was done with the standard six-step procedure outlined by CHARMM-GUI, which consists of two steps of NVT ensemble (constant number of particles, constant volume, and temperature) followed by four steps of NPT ensemble (constant number of particles, constant pressure, and temperature) equilibration. The timestep used in the first three steps was 1 fs, while the last three steps of the equilibration scheme used the 2 fs timestep. Restraints to certain components in the system were relaxed during the subsequent stages. The equilibration procedure also consisted of 3000 steps of energy minimization using the steepest descent and the adaptive basis Newton–Raphson method.

Each system was exposed to a long equilibration phase of at least 100 ns. All simulations were carried out on GPUs with the CUDA version of the NAMD [33] (ver. 2.13) MD software suite. The CHARMM36 [34] force field was used, which includes parameters for protein, membrane, water, ions, and galactose. Missing parameters for inhibitors were derived using the Paramchem [35] server while the partial atomic charges were further refined by ab-initio calculation performed by Gaussian suit of programs [27]. All production simulations used the NPT ensemble and their total length was between 0.7 μs and 1.2 μs. The temperature was held constant (303.15 K) using the Langevin thermostat with a dampening constant of 1 ps^−1^ [36]. The pressure was also held constant (1.01325 bar) by the use of the Nose–Hoover Langevin piston for pressure control [37,38]. The timestep of the production simulations was 2 fs, and the SHAKE [39] algorithm was used for hydrogen atoms. The cutoff for nonbonded interactions was set to 12 Å, electrostatic interactions were calculated using the Particle Mesh Ewald method [40].

### 4.4. Determining Water Permeation Events

The quantification of water permeation was performed by counting only the complete permeation events of all water molecules crossing the protein channel from one side to the other, excluding the eventual pathways at the protein-membrane interface. In order to exclude crossings due to PBC-wrapping, we defined two planes parallel to the membrane, separated by Δ = 25 Å and positioned symmetrically one on each side of the membrane mirror plane. In addition, we introduced two external slabs of thickness δ = 5 Å on both sides indicating inner and outer compartment. If $z_{i}\left(t^{*}\right)$ is a *z* component of the CoM of *i*-th water molecule and $t^{*}$ is the simulation time at which the molecule is found in the inner compartment fulfilling a condition $-\Delta /2-\delta <z_{i}\left(t^{*}\right)<-\Delta /2$, then the molecule successfully permeates through the protein if it is detected in the outer compartment as some latter time τ, such that $\Delta /2<z_{i}\left(t^{*}+\tau \right)<\Delta /2+\delta$. The same principle applies for the reverse direction.

### 4.5. Principal Component Analysis

PCA is an efficient method for decomposing protein dynamics into collective (essential) directions of motion, termed as principal components [41,42].

Principal components are obtained by solving the secular equation

(1) $$\det\left(C-\lambda I\right)=0\;\equiv \;E^{T}CE=\lambda .$$

Here, C is the covariance matrix of atomic fluctuations, $C_{ij}=\langle \left(r_{i}-\langle r_{i}\rangle \right)\left(r_{j}-\langle r_{j}\rangle \right)\rangle$, λ is a diagonal matrix, $\Delta_{ij}\lambda_{i}$, with eigenvalues $\lambda_{i}$ and $E=(e_{1},e_{2},\ldots )$ are the associated eigenvectors which correspond to principal components. 〈〉 denotes the ensemble average (time average in case of MD) and $r_{i}$ is an instantaneous position coordinate of an *i*-th object, which could be an atom or CoM of a selected group of atoms. Individual $\lambda_{i}$ can be expressed as the mean square of collective mode coordinate fluctuation along the vector $e_{i}$.

To allow further analysis, we may define the effective force constant of the mode, $\kappa_{i}=k_{B}T/\lambda_{i}$, which we may also call mode stiffness. Alternatively, we can introduce mode softness or mode flexibility as reciprocal mode stiffness, $\sigma_{i}=\kappa_{i}^{-1}=\lambda_{i}/\left(k_{B}T\right)$, which is also proportional to the amplitude of the mode fluctuations. Flexibility is meaningful only for vibrational modes. However, especially in low-dimensional systems, some mixing between rotational and vibratory modes may persist because the pure rigid rotation can not be easily completely removed from the trajectory. We introduce an additional weighting factor $w_{vi}$ indicting a pure vibrational nature of the *i*-th mode, which can readily be calculated from the total kinetic energy of the mode has given that eigenvector components describe bead displacements in a unit of time. Now, the overall protein flexibility σ is defined by the sum over all PCA modes while taking into account only vibrational modes as:

(2) $$\sigma =\sum_{i}\sigma_{i}=\frac{1}{k_{B}T}\sum_{i}\lambda_{i}w_{vi}.$$

In this view, higher flexibility indicates the presence of modes with more enhanced thermal fluctuations. It is generally accepted that the low-frequency modes are functionally relevant, but that does not imply that the lowest frequency mode corresponds exactly to the functional mode. The latter is more likely to emerge as a superposition of several PCA modes. In search of the existence of a specific functional mode and in order to make the comparison between different systems more consistent, we prefer to define bead displacements in a form of a normalized (projecting) vector P having the same dimension as eigenvectors e. If P encodes a specific type of motion, then the flexibility of this mode is

(3) $$\sigma_{P}=\sum_{i}\sigma_{i}=\frac{1}{k_{B}T}\sum_{i}\lambda_{i}w_{vi}\left(P\cdot e_{i}\right).$$

All PCA-based codes and scripts are home-made and are available to interested readers upon request to the corresponding author.

## 5. Conclusions

In our study, we have identified a strong dependence of inhibitor binding on the rigidification of the internal motion of the two primary domains (the bundle and the hash domain) of the cotransporter in addition to the constriction of the water channel. We have also identified a stiffening of the functional modes resembling the rocking-bundle type motion, a motion critical for the conformation switch, as a result of the binding of the inhibitor molecules. The rocking-bundle type of motion is characteristic of LeuT-fold cotransporters. Thus, it appears that the behavior of the protein structure at the domain level plays as important a role in water transport as local aspects described by the geometry of the gating regions. The mechanism by which inhibitors rigidify the SGLT1 structure is by allosterically increasing the interaction between the bundle and the hash domain. In this regard, our results highlight a property of the transporter flexibility as an important aspect that should be considered in the development of new inhibitors of water transport. In this work, we also demonstrate the applicability of the novel computational approach as a tool for assessing the structural flexibility of a transporter as a determinant of molecular transport.

## Figures and Tables

**Table 1:** Time-averaged interaction energies between SGLT1 and bound ligands (second column) and between bundle and hash domain of the SGLT1 (last column) during simulations. In the third column are given the binding free energies calculated according to the MMPBSA method.

Ligand	$E_{\mathrm{int}}$(lig.:prot.) [kcal/mol]	$\Delta G_{\mathrm{MMPBSA}}$(lig.:prot.) [kcal/mol]	$E_{\mathrm{int}}$(bundle:hash) [kcal/mol]
phlorizin	−80.5 ± 6.6	−29.5 ± 4.7	−95.9 ± 9.1
sotagliflozin	−89.5 ± 7.1	−42.5 ± 5.2	−101.6 ± 11.5
mizagliflozin	−112.9 ± 9.3	−47.2 ± 6.8	−99.8 ± 10.9
galactose	−26.2 ± 6.4	−4.4 ± 2.1	−68.4 ± 4.5
no ligand	/	/	−56.4 ± 11.9

**Table 2:** Half-maximal inhibitory concentration values IC${}_{50}$ for inhibiting sugar transport in SGLT1 [22].

Inhibitor	IC${}_{50}$ [nmol/L]
phlorizin	400
sotagliflozin	36
mizagliflozin	27

## Data Availability

All data are available upon reasonable request.

## Supplementary Materials

The following supporting information can be downloaded at: https://www.mdpi.com/article/10.3390/molecules28145295/s1, Selection of residues based on the wettability criteria. Displayed residue number residue name in 3-letter code and an identifier if the residues is part of the bundle (B), hash (H), gating (G) domains or other (O). Figure S1: RMSD for the core region of SGLT1 as a function of simulation time of the OF cryoEM simulation against the time averaged structure of the homoloy model (red curve) and RMSD of the homology model simulation against the time averaged OF cryoEM structure (blue curve).

## Abbreviations

The following abbreviations are used in this manuscript:

SGLT	Sodium glucose cotransporters
MD	Molecular Dynamics
PCA	Principal Component Analysis
CoM	Center of Mass
