# Supplementary material for: Influence of SGLT1 Sugar Uptake Inhibitors on Water Transport
Source: Molecules. 2023 Jul 8;28(14):5295. doi: 10.3390/molecules28145295 (PMC10385929; doi:10.3390/molecules28145295)
Supplement: Supplementary file 1 [file molecules-28-05295-s001.zip › molecules-2425670-supplementary.pdf]

Supplementary information

Influence of SGLT1 sugar uptake inhibitors on water transport

Marko Sever, Franci Merzel

Selection of residues based on the wettability criteria

Displayed residue number residue name in 3-letter code and an identifier if the residues is part of the bundle (B), hash (H), gating (G) domains or other (O).

Out-apo	Phlorizin	Sotagliflozin	Mizagliflozin
77 SER B	77 SER B	77 SER B	78 ASN B
78 ASN B	78 ASN B	78 ASN B	80 GLY B
80 GLY B	82 GLY B	81 SER B	81 SER B
81 SER B	86 GLY B	82 GLY B	82 GLY B
82 GLY B	100 GLY B	83 HSD B	83 HSD B
83 HSD B	149 SER H	149 SER H	101 PHE B
86 GLY B	156 THR H	161 ASP H	105 ALA B
99 GLY B	160 ALA H	180 ALA H	156 THR H
149 SER H	161 ASP H	278 GLY B	157 LYS H
156 THR H	278 GLY B	284 SER B	159 SER H
157 LYS H	283 MET B	289 TRP B	161 ASP H
159 SER H	284 SER B	293 THR B	282 GLY B
160 ALA H	287 THR B	384 SER H	437 SER O
161 ASP H	294 ASP B	392 SER H	454 ASP G
282 GLY B	389 SER H	393 SER H	458 SER G
284 SER B	392 SER H	396 SER H	519 THR G
287 THR B	393 SER H	453 PHE G	
290 TYR B	437 SER H	454 ASP G	
294 ASP B	453 PHE G	458 SER G	
325 MET B	454 ASP G	516 ASN O	
392 SER H	457 GLN G	517 CYS O	
393 SER H	458 SER G	519 THR O	
396 SER H	496 GLY O		
437 SER H	518 PRO O		
453 PHE G			
454 ASP G			
455 TYR G			
457 GLN G			
458 SER G			
461 SER G			
468 ALA O			
502 THR O			
503 GLU O			
509 GLY O			
510 SER O			
518 PRO O			

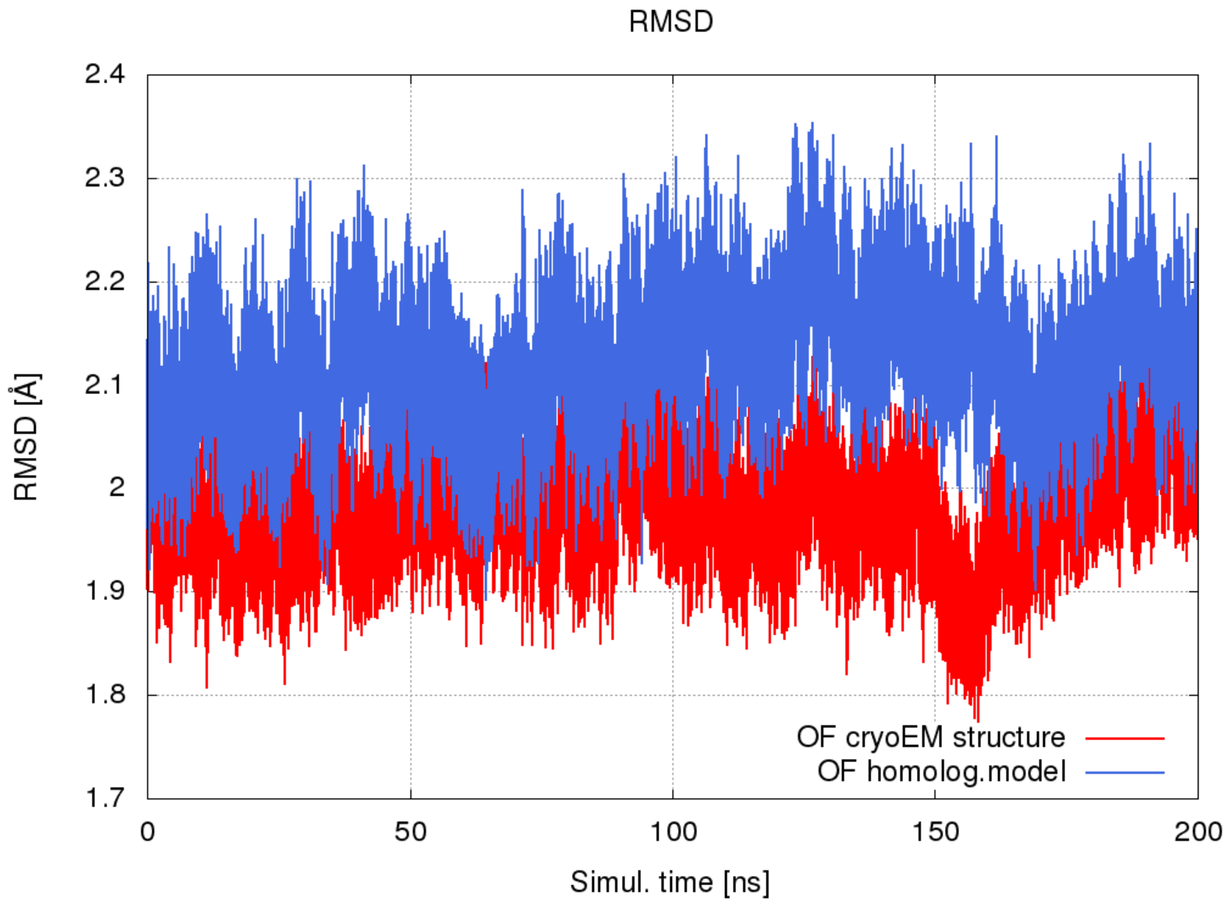

Figure S1. RMSD for the core region of SGLT1 as a function of simulation time of the OF cryoEM simulation against the time averaged structure of the homoloy model (red curve) and RMSD of the homology model simulation against the time averaged OF cryoEM structure (blue curve).
